# Molecular Noninvasive Diagnosis of Hepatocellular Carcinoma Using Microsatellite Instability

**DOI:** 10.31557/APJCP.2021.22.10.3337

**Published:** 2021-10

**Authors:** Samah Mamdouh, Tarek Aboushousha, Amr Abdelraouf, Hussam Hamdy, Mohamed Seleem, Hanem Hassan

**Affiliations:** 1 *Departement of Biochemistry and Molecular Biology, Theodor Bilharz Research Institute, Giza, Egypt. *; 2 *Department of Pathology, Theodor Bilharz Research Institute, Egypt. *; 3 *HBP Surgery, National Hepatology and Tropical Medicine Research Institute, Egypt. *; 4 *Department of Surgery, Theodore Bilharz Research Institute, Giza, Egypt. *; 5 *Department of Hepato-Pancreatic-Biliary Surgery, National Hepatology and Tropical Medicine Research Institute, Cairo, Egypt. *

**Keywords:** HCC, microsatellite instability, noninvasive

## Abstract

**Objective:**

Hepatocellular carcinoma (HCC) accounts for more than 80% of primary liver cancers. Moreover, in the next 10 years, more than one million patients are expected to die from liver cancer as estimated by the World Health Organization. The aim of the present study is to define the microsatellite phenotype in the blood, tumor and nontumor tissue samples from hepatocellular carcinoma cases to develop a simple non-invasive method for diagnosis and detection of the disease.

**Methods:**

A total of 100 patients with histologically-proven HCC were enrolled in this study, blood samples and tissue specimens from tumor and nontumor tissue were obtained from each patient. DNA was extracted and microsatellite instability MSI status was determined by polymerase chain reaction (PCR) using 5 mononucleotide and 5 dinucleotide repeats.

**Results:**

Among the 100 HCC tumors analyzed, (8%) considered as displaying a typical MSI-H phenotype as defined by instability in at least 3 of the 10 repeats analyzed, (61%) tumors displayed MSI-L and (31%) displayed MSS while in plasma the instability was (40%) for MSI-H, (44%) for MSI-L and (16%) for MSS.

**Conclusion:**

our findings could point to the achievement that HCC patients could be diagnosed by MSI analysis using blood sample as non-invasive way and this conclusion achieved our aim as the study shows impressive and promising results.

## Introduction

Hepatocellular carcinoma (HCC) is one of the most prevalent malignancies in the world and the third most common cause of cancer-related deaths. It is a high incidence disease in Egypt with a poor prognosis and survival (Balogh et al., 2016). In Egypt, it represents the fourth common cancer (Akinyemiju et al., 2017) and the fifth leading cause of cancer worldwide in men and the seventh in women (Goumard et al., 2017). 

Several studies have investigated whether HCC might be associated with microsatellite instability (MSI), a well-known oncogenic pathway for other cancers such as colon and gastric cancers (Macdonald et al., 1998).

Microsatellites (MS) are tandem repeats of short DNA sequences, abundant throughout the human genome (Samah et al., 2015). Owing to their high mutation rates, MS have been widely used as polymorphic markers in population genetics and forensics. MSI is a hypermutator phenotype that occurs in tumors with impaired DNA mismatch repair (MMR) and is characterized by widespread length polymorphisms of MS repeats due to DNA polymerase slippage (Cortes et al., 2017).

The distribution characteristics are different from 15 to 65 nucleotides tandem repeats of small satellite DNA, which is mainly located near the ends of chromosomes. MS are widely distributed and is mostly located near the coding region and may be located others region like intron or non-coding region. Each MS specific site is composed of two parts: the central core and the peripheral flanks, and the specificity of MS is mainly due to the change in the number of core repeating units (Li et al., 2020).

Microsatellite instability seems to be a rare event in hepatocarcinogenesis and might actually be associated with the progression of hepatocellular carcinoma in which the liver is often the site of chronic hepatitis or cirrhosis (Chiappini et al., 2004).

The mechanism of MS generation is generally believed to be DNA slippage in the process of replication, or mismatch of the basic group of slippage strand and complementary strand in the process of DNA replication and repair, resulting in one or more of the repeating units missing or insert. The normal tissue DNA repair system, called mismatch repair, can correct in the process of DNA replication errors. However, due to the lack of MMR genes in tumor cells or defects in the process of replication repair, the possibility of gene mutation is increased (lower et al., 2018). It can be seen that MSI is an important factor in the occurrence and development of tumors (Ionov et al., 1993; Aaltonen et al., 1993).

The aim of the present study is to define the MSI phenotype in HCC and to detect the occurrence of 5 monomicrosatellite instability markers and 5 dimicrosatellite instability markers in the blood, tumor and adjacent nontumor tissue samples from HCC cases and comparing the results to the clinicopathological data of the patients to develop a simple non-invasive method for diagnosis and detection of the disease.

## Materials and Methods

*Patients and samples*

A total of 100 patients with histologically-proven HCC were surgically resected, Five milliliter venous blood samples and malignant and corresponding peri-malignant liver tissue samples were obtained from National Hepatology and Tropical Medicine Research Institute (NHTMRI), and Theodor Bilharz Research Institute (TBRI), Cairo, Egypt. Blood samples from 25 healthy volunteers were obtained as controls. This study was carried out in full accordance with the Helsinki Declaration of 1975, as revised in 1983, and was approved by the Ethics Committee of Theodor Bilharz Research Institute and by National Hepatology and Tropical Medicine Research Institute. A written informed consent was obtained from each participant, in accordance with the institutional guidelines.

The histology was assessed just after surgery by a pathologist and then checked by a second pathologist.

*Processing of blood samples*

Five milliliter of blood was aseptically withdrawn from each patient via venipuncture into EDTA tube then centrifuged at 5,000 g for 10 min and plasma was stored at -20^o^C until analysis. 

*Processing of tumor and adjacent non-tumor tissue samples*

Liver tissues obtained were divided and samples used for molecular techniques were stored at -80ºC until use. The remaining tissue samples were preserved in 10% formalin until processed for pathological examination. 

*DNA Extraction and Molecular analysis for MSI status*

Total DNA was extracted from the homogenized tissue samples and plasma using the Abott kit. (Abbott Park, Illinois, U.S.A), according to the manufacturer’s protocol. The DNA samples were coded and stored at -20°C until analysis.

MSI analysis was carried out using 5 quasi-monomorphic mononucleotide repeats markers (BAT25, BAT26, NR21, NR22, NR24) and 5 highly polymorphic dinucleotide repeat markers (D-8S298, D-8S1771, D-8S277, D-2S123 and D-17S250). DNA was amplified using Taq DNA Polymerase (Qiagen), starting with an initial denaturation step at 95^o^C for 5 minutes followed by 40 cycles each cycle started with denaturation step at 95^o^C for 30 sec, annealing at 55˚C for 30 sec and extension at 72^o^C for 1 min, with a final extension at 72^o^C for 10 min. The reaction was performed in a 25 μl volume and the amplified PCR products were electrophoresed on 12% PAGE polyacrylamide gel electrophoresis (3 h 150 V) and visualized by ethidium bromide staining. The gel image was analyzed using Cleaver micro DOC gel documentation system. Purified PCR products were run on an ABI PRISM 3100 Genetic Analyzer (Applied Biosystems), allelic sizes were defined using the GeneMapper Software (Applied Biosystems).

*Statistical analysis*

The data were analysed using Microsoft Excel 2016 and statistical package for social science ‘IBM SPSS Statistics for Windows, version 26 (IBM Corp., Armonk, N.Y., USA)’. Continuous normally distributed variables were represented as mean±SD. with 95% confidence interval, while nonnormal variables were summarized as median with 25 and 75 percentile, the frequencies and percentage for categorical variables were used; a P value < 0.05 was considered statistically significant. To compare the means of normally distributed variables between groups, the Student’s t test was performed, and Mann-Whitney U test was used in non-normal variables. χ^2^ test or Fisher’s exact test was used to determine the distribution of categorical variables between groups. The risk assessment OR (95% C.I) was done by using the binary logistic regression analysis.

## Results

Microsatellite instability was examined using five monomorphic microsatellite markers and five polymorphic repeats were amplified from both the tumor and nontumor liver DNAs and the corresponding blood from 100 HCC patients, their age was with an average of 49.2±9.3 years. The demographic and clinical characteristics are illustrated in (Table 1). Blood samples from 25 healthy volunteers were also obtained as a control. Each PCR amplification was repeated twice. A tumor was defined as MSI-H when shifts occurred in at least 30% of assessable markers, tumors in which < 30% of assessable markers were altered were classified as MSI-L. Tumors were considered to be MSS if none of the markers showed instability (Boland et al., 1998). 

The percentages of the positive results for the studied monomicrosatellite markers (BAT-25, BAT-26, NR-21, NR-22 and NR-24) in tissues were (0%, 22%, 16%, 2% and 5) and in plasma the results were (23%, 30%, 31%, 28%, and 31) respectively, while for dimicrosatellites (D-8S1771, D-8S277, D-8S298, D-2S123 and D-17S250) the results were (3%, 8%, 0%, 38% and 34) in tissues and in plasma the results were (23%, 34%, 25%, 41% and 34) respectively. Regarding to the general scale of instability (Boland, etal., 1998), the studied MS showed 31% for MSS, 61% for MSI-L and only 8% for MSI-H in tissues while in plasma the instability was 16% for MSS, 44% for MSI-L and 40% for MSI-H. 

By classifying the studied cases according to the source of viremia, the demographic and clinical characteristics were shown in (Table 1). In addition, it was found that 7 MS have a clear instability in cases of HBV infection than HCV as follows, NR-21 showed significant instability in HBV than HCV patients (P. value =0.03) and OR (95% C.I) = 1.46 (0.94-2.28) , NR-22 (P. value =0.047) and OR (95% C.I) = 0.20 (0.14 -0.30), NR-24 (P. value <0.001) and OR (95% C.I) = 0.18(0.12 -0.28), D-8S1771 (P. value = 0.01) and OR (95% C.I) = 0.20 (0.13 -0.29), D-8S277 (P. value <0.001) and OR (95% C.I) = 0.15 (0.09 -0.25), D-2S123 (P. value =0.02) and OR (95% C.I) = 1.30 (1.01 -1.67), and D-17S250 (P. value =0.02) and OR (95% C.I) = 1.31(1.00 -1.71) (Table 2). 

Interestingly, a percentage of 36.4 was observed representing MSI-H in cases of HBV infection (P. value <0.001) and OR (95% C.I) = 0.13 (0.02 - 0.99), while 37.2% was MSS in cases of HCV infection (P. value <0.001) and OR (95% C.I) = 14.5 (3.46 - 60.77).

HBV cases showed significantly higher percentage of MSI-H positivity than HCV cases (P. value <0.001). Also, higher tumor grades with solid and acinar/solid pattern showed high percentage of MSI-H positive cases (Table 2). On the contrary, low grades of hepatitis activity showed higher percentage of MSI-H cases.

MSI-L and MSI-H showed no significant influence on the distribution of steatosis positive and negative cases. 

In plasma samples, 4 MS were observed to have a clear instability in cases of HBV infection than HCV as follows, BAT-26 showed significant instability in HBV than HCV patients (P. value =0.001) and OR (95% C.I) = 0.36 (0.17 - 0.74), D-8S277 (P. value <0.001) and OR (95% C.I) = 0.19 (0.08 - 0.45), D-2S123 (P. value <0.001) and OR (95% C.I) = 0.20 (0.08 - 0.51), and D-17S250 (P. value <0.001) and OR (95% C.I) = 0.15 (0.06 - 0.38) (Table 3).

Interestingly, a percentage of 100 was observed representing MSI-H in cases of HBV infection (P. value <0.001) and OR (95% C.I) = 0.11 (0.04 – 0.37), while 56.4% was MSI-L in cases of HCV infection.

Regarding the stage of disease, the demographic and clinical characteristics were as shown in (Table 4). In addition, it was found that only one MS (D-17S250) has a clear instability in cases of fibrosis grade than cirrhosis (P. value =0.001) and OR (95% C.I) = 0.25 (0.10 -0.60) in tissues (Table 5). 

In plasma samples, 6 MS were observed to have a clear instability in cases of fibrosis grade than cirrhosis as follows, BAT-25 showed significant instability (P. value <0.001) and OR (95% C.I) = 0.40 (0.27 - 0.61), BAT-26 (P. value =0.01) and OR (95% C.I) = 0.52(0.33 - 0.82), NR-21 (P. value <0.001) and OR (95% C.I) = 0.50 (0.32 - 0.78), NR-22 (P. value =0.03) and OR (95% C.I) = 0.58 (0.37 - 0.92), D-8S1771 (P. value <0.001) and OR (95% C.I) = 0.40 (0.27 - 0.61), and D-8S298 (P. value <0.001) and OR (95% C.I) = 0.50 (0.32 - 0.78) (Table 6).

Interestingly, a percentage of 51.7 was observed representing MSI-L was in cases of cirrhosis (P. value =0.01) and OR (95% C.I) =2.4 (1.248 - 4.557).

According to the association study between MSI-H in tissues with the studied parameters, it was found that there is a significant association with the following parameters, low albumin (P. value <0.001), high level of AFP (P. value <0.01), higher number of masses (P. value <0.01), larger tumor size (P. value =0.04), advanced tumor grade II (P. value =0.04) and grade III (P. value <0.01), solid pattern (P. value =0.02) and acinar/solid pattern (P. value =0.04), HAI A1 (P. value <0.01), Splenomegaly (P. value =0.03), and in patients with lower limb edema (P. value =0.04) (Table 7). 

While, in plasma samples, a significant association was found with high level of ALT (P. value <0.001), low albumin (P. value =0.01), high level of AFP (P. value <0.01), higher number of masses (P. value <0.001), larger tumor size (P. value <0.001), advanced tumor grade III (P. value <0.001), Acinar/Solid pattern (P. value <0.001), HAI A3 (P. value <0.001), Hepatomegaly (P. value <0.001), Ascites (P. value <0.001), Splenomegaly (P. value <0.001), and in patients with lower limb edema (P. value <0.001) (Table 7). 

Finally, we can deduce the results of association analysis as follows, as regards the gross and histopathological differences between the MSI-L & MSI-H groups, it was found that the number of tumor masses and the mean tumor size were higher in the MSI-H group compared to the MSI-L group with (P. value <0.01 and <0.04) respectively (Table 7).

Molecular Evolutionary Genetic Analysis (MEGA-X) software (version 10.2.4) was used to detect the nucleotide insertion, deletion or substitution by alignment.

**Figure 1:**
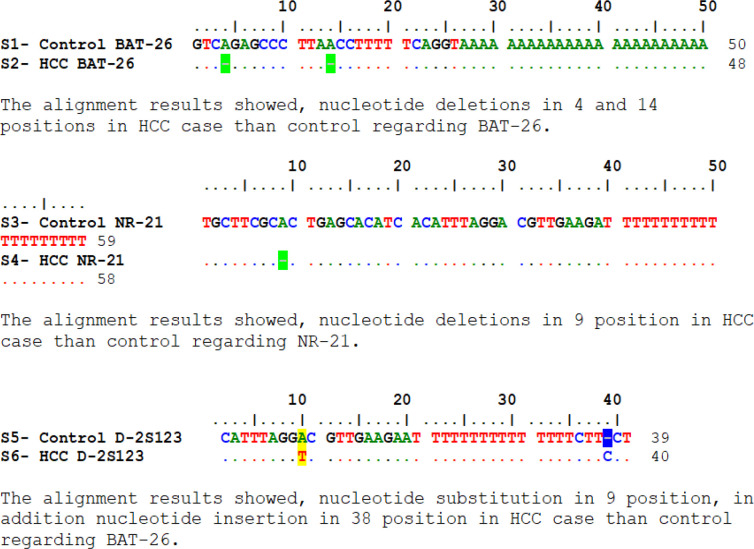
Results of Sequence Alignment of HCC DNA with Control

Many assays gave nucleotide shift on plasma monomicrosatellite markers specially BAT-26 and NR-21 as nucleotide deletions and on dimicrosatellite D-2S123 as single nucleotide polymorphism and nucleotide insertion. These results confirm the importance and the value of using blood samples from patients for diagnosis as non-invasive method to detect the disease and this achieves the aim of the study (Figure 1).

**Table 1:** Demographic and Clinical Characteristics Data in General and Regarding the Source of Viremia

		Total	HBV	HCV	P. Value
		N=100	N=22	N=78	
Age		49.2±9.3	46.9±7.2	49.8±9.8	0.2
Sex	Female	20 (20.0)	9 (40.9)	11(14.1)	0.01*
	Male	80 (80.0)	13 (59.1)	67(85.9)	
ALT		72.3±30.7	87.7±41.3	67.9±25.7	0.01*
AST		77.5±36.2	93.1±51.5	73.0±29.4	0.02*
Alb		1.9±1.06	1.6±1.1	2.0±1.0	0.1
Bilirubin		2.7±1.09	2.9±1.4	2.7±1.0	0.3
AFP		97.5 (50.0- 600.0)	290.0 (25.0- 2300.0)	90.0 (50.0- 362.5)	0.03*
S-creatinine		1.1±0.4	1.2±0.4	1.1±0.4	0.2
No. of masses		1.6±0.9	2.2±1.2	1.4±0.8	0.001**
Tumor size		2.5 (1.3 – 3)	15.3 (0.7- 110.0)	3.5 (1.3- 5.3)	0.04*
Tumor Grade	I	60 (60.0)	9 (40.9)	51 (65.4)	0.001**
	II	20 (20.0)	3 (13.6)	17 (21.8)	0.05*
	III	20 (20.0)	10 (45.5)	10 (12.8)	0.001**
Pattern	Acinar	45 (45.0)	2 (9.1)	43 (55.1)	0.001**
	Solid	38 (38.0)	12 (54.5)	26 (33.3)	0.01*
	Acinar/Solid	17 (17.0)	8 (36.4)	9 (11.5)	0.001**
Steatosis		0.04 (0.01- 0.08)	0.02 (0.01- 0.13)	0.03 (0.02- 0.2)	0.05*
Stage	Fibrosis	40 (40.0)	8 (36.4)	32 (41.0)	0.4
	Cirrhosis	60 (60.0)	14 (63.6)	46 (59.0)	
HAI	A1	21 (21.0)	3 (13.6)	18 (23.1)	0.03*
	A2	65 (65.0)	15 (68.2)	50 (64.1)	0.4
	A3	14 (14.0)	4 (18.2)	10 (12.8)	0.1
Hepatomegaly	Positive	46 (46.0)	19 (86.4)	27 (34.6)	0.001**
Ascites	Positive	59 (59.0)	16 (72.7)	43 (55.1)	0.1
Splenomegaly	Positive	52 (52.0)	22 (100.0)	30 (38.5)	0.001**
Edema Lower Limbs	Positive	69 (69.0)	18 (81.8)	51 (65.4)	0.1

Age, ALT, AST, Alb, Bilirubin, S-creatinine, and No. of masses are represented as Mean and SD; the data were analyzed by t test, But Alpha feto-protein, Tumor size, and Steatosis are represented as Median and Interquartile Range (IR) (25% -75); the data were analyzed by Mann-Whitney U test., While Sex, Grade, Pattern, Stage, HAI, Hepatomegaly, Ascites, Splenomegaly, and Edema Lower Limbs are represented as Frequency and percent; the data were analyzed by X^2 ^test. * P value <0.05 is significant, ** P value <0.01 is highly significant.

**Table 2:** Studied Microsatellites Regarding the Source of Viremia with the Risk Assessment in HCC Tissues

		Source of viremia			Risk assessment		
		HBV	HCV	P. Value	OR	95% C.I	P. Value
		N=22 (%)	N=78 (%)				
BAT-25	Stable	22 (100.0)	78 (100.0)	N.A	N.A		
	Instable	0 (0.0)	0 (0.0)				
BAT-26	Stable	17 (77.3)	61 (78.2)	0.6	1.01	0.78 - 1.31	0.9
	Instable	5 (22.7)	17 (21.8)				
NR-21	Stable	15 (68.2)	69 (88.5)	0.03*	1.46	0.94 - 2.28	0.02*
	Instable	7 (31.8)	9 (11.5)				
NR-22	Stable	20 (90.9)	78 (100.0)	0.047*	0.2	0.14 -0.30	0.01*
	Instable	2 (9.1)	0 (0.0)				
NR-24	Stable	17 (77.3)	78 (100.0)	<0.0001**	0.18	0.12 -0.28	0.001**
	Instable	5 (22.7)	0 (0.0)				
D-8S1771	Stable	19 (86.4)	78 (100.0)	0.01*	0.2	0.13 -0.29	0.01*
	Instable	3 (13.6)	0 (0.0)				
D-8S277	Stable	14 (63.6)	78 (100.0)	<0.0001**	0.15	0.09 -0.25	0.001**
	Instable	8 (36.4)	0 (0.0)				
D-8S298	Stable	22 (100.0)	78 (100.0)	NA	NA		
	Instable	0 (0.0)	0 (0.0)				
D-2S123	Stable	9 (40.9)	53 (67.9)	0.02*	1.3	1.01 -1.67	0.02*
	Instable	13 (59.1)	25 (32.1)				
D-17S250	Stable	10 (45.5)	56 (71.8)	0.02*	1.31	1.00 -1.71	0.02*
	Instable	12 (54.5)	22 (28.2)				
Instability	MSS	2 (9.1)	29 (37.2)	0.001**	14.5	3.46 - 60.77	0.001**
	MSIL	12 (54.5)	49 (62.8)	0.09	0.282	0.059 - 1.348	0.1
	MSIH	8 (36.4)	0 (0.0)	0.001**	0.13	0.02 - 0.99	0.05*

Studied microsatellites are represented as Frequency and percent; the data were analyzed by X^2^ test. OR; Odd Ratio, C.I; Confidence Interval, P value calculated depend on logistic regression analysis. * P value <0.05 is significant, ** P value <0.01 is highly significant.

**Table 3:** Studied Microsatellites Regarding the Source of Viremia with the Risk Assessment in Plasma

		Source of viremia			Risk assessment		
		HBV	HCV	P. Value	OR	95% C.I	P. Value
		N=22 (%)	N=78 (%)				
BAT-25	Stable	17 (77.3)	60 (76.9)	0.6	1.02	0.42 - 2.45	0.9
	Instable	5 (22.7)	18 (23.1)				
BAT-26	Stable	10 (45.5)	60 (76.9)	0.001**	0.36	0.17 - 0.74	0.01*
	Instable	12 (54.5)	18 (23.1)				
NR-21	Stable	12 (54.5)	57 (73.1)	0.08	0.54	0.26 - 1.11	0.1
	Instable	10 (45.5)	21 (26.9)				
NR-22	Stable	14 (63.6)	58 (74.4)	0.2	0.68	0.32 - 1.44	0.3
	Instable	8 (36.4)	20 (25.6)				
NR-24	Stable	12 (54.5)	57 (73.1)	0.08	0.54	0.26 - 1.11	0.1
	Instable	10 (45.5)	21 (26.9)				
D-8S1771	Stable	15 (68.2)	62 (79.5)	0.2	0.64	0.30 - 1.38	0.3
	Instable	7 (31.8)	16 (20.5)				
D-8S277	Stable	6 (27.3)	60 (76.9)	0.001**	0.19	0.08 - 0.45	0.001**
	Instable	16 (72.7)	18 (23.1)				
D-8S298	Stable	19 (86.4)	56 (71.8)	0.1	2.11	0.68 - 6.54	0.2
	Instable	3 (13.6)	22 (28.2)				
D-2S123	Stable	5 (22.7)	54 (69.2)	0.001**	0.2	0.08 - 0.51	0.001**
	Instable	17 (77.3)	24 (30.8)				
D-17S250	Stable	5 (22.7)	61 (78.2)	0.001**	0.15	0.06 - 0.38	0.001**
	Instable	17 (77.3)	17 (21.8)				
Instability	MSS	0 (0.0)	16 (20.5)	-	-	-	-
	MSIL	0 (0.0)	44 (56.4)	-	-	-	-
	MSIH	22 (100.0)	18 (23.1)	0.001**	0.11	0.04 – 0.37	0.001**

Studied microsatellites are represented as Frequency and percent; the data were analyzed by X^2^ test. OR; Odd Ratio, C.I; Confidence Interval, p value calculated depend on logistic regression analysis. * P value <0.05 is significant, ** P value <0.01 is highly significant.

**Table 4:** Demographic and Clinical Characteristics Data Regarding the Stage of Disease

		Fibrosis	Cirrhosis	P. Value
		N=40	N=60	
Age		52.3±9.7	47.1±8.5	0.01*
Sex	Female	7 (17.5)	13 (21.7)	0.4
	Male	33 (82.5)	47 (78.3)	
ALT		77.9±34.4	68.6±27.7	0.2
AST		83.7±41.2	73.3±32.0	0.2
Alb		1.6±0.9	2.1±1.1	0.02*
Bilirubin		2.6±1.0	2.8±1.1	0.4
Alpha feto-protein		360.0 (77.5- 2300.0)	75.0 (40.0- 290.0)	0.001**
S-creatinine		1.3±0.4	1.0±0.4	0.04*
No. of masses		1.4±0.7	2.0±1.2	0.01*
Tumor size		1.5 X 2.5 (1 X1.1 – 3 X7)	1X3.8 (1X1.3 - 2X1.5)	0.1
Tumor Grade	I	8 (20.0)	14 (23.3)	0.4
	II	32 (80.0)	46 (76.7)	0.4
	III	24 (60.0)	36 (60.0)	NA
Pattern	Acinar	18 (30.0)	2 (5.0)	0.001**
	Solid	6 (10.0)	14 (35.0)	0.001**
	Acinar/Solid	24 (40.0)	21 (52.5)	0.01*
Steatosis		0.1 (0.03- 0.6)	0.02 (0.02- 0.03)	0.001**
HAI	A1	0 (0.0)	21 (35.0)	0.001**
	A2	26 (65.0)	39 (65.0)	NA
	A3	14 (35.0)	0 (0.0)	0.001**
Hepatomegaly	Positive	27 (67.5)	19 (31.7)	0.001**
Ascites	Positive	28 (70.0)	31 (51.7)	0.05*
Splenomegaly	Positive	23 (57.5)	29 (48.3)	0.2
Edema Lower Limbs	Positive	26 (65.0)	43 (71.7)	0.3

Age, ALT, AST, Alb, Bilirubin, S-creatinine, and No. of masses are represented as Mean and SD; the data were analyzed by t test, But Alpha feto-protein, Tumor size, and Steatosis are represented as Median and Interquartile Range (IR) (25% -75); the data were analyzed by Mann-Whitney U test., While Sex, Grade, Pattern, HAI, Hepatomegaly, Ascites, Splenomegaly, and Edema Lower Limbs are represented as Frequency and percent; the data were analyzed by X2 test. * P value <0.05 is significant, ** P value <0.01 is highly significant.

**Table 5:** Studied Microsatellites Regarding the Stage of Disease with the Risk Assessment in HCC Tissues

		Stage of disease			Risk assessment		
		Fibrosis	Cirrhosis	P. Value	OR	95% C.I	P. Value
		N=40 (%)	N=60 (%)				
BAT-25	Stable	40 (100.0)	60 (100.0)	NA	NA		
	Instable	0 (0.0)	0 (0.0)				
BAT-26	Stable	33 (82.5)	45 (75.0)	0.3	1.57	0.58 -4.29	0.4
	Instable	7 (17.5)	15 (25.0)				
NR-21	Stable	34 (85.0)	50 (83.3)	0.5	1.13	0.38 -3.41	0.8
	Instable	6 (15.0)	10 (16.7)				
NR-22	Stable	38 (95.0)	60 (100.0)	0.2	0.39	0.30 -0.50	0.08
	Instable	2 (5.0)	0 (0.0)				
NR-24	Stable	40 (100.0)	55 (91.7)	0.07	0.58	0.49 -0.69	0.06
	Instable	0 (0.0)	5 (8.3)				
D-8S1771	Stable	40 (100.0)	57 (95.0)	0.2	0.59	0.50 -0.69	0.2
	Instable	0 (0.0)	3 (5.0)				
D-8S277	Stable	38 (95.0)	54 (90.0)	0.3	2.11	0.40 -11.03	0.4
	Instable	2 (5.0)	6 (10.0)				
D-8S298	Stable	100.00%	100.00%	NA	NA		
	Instable	0 (0.0)	0 (0.0)				
D-2S123	Stable	21 (52.5)	41 (68.3)	0.08	0.51	0.22 -1.17	0.1
	Instable	19 (47.5)	19 (31.7)				
D-17S250	Stable	19 (47.5)	47 (78.3)	0.001**	0.25	0.10 -0.60	0.001**
	Instable	21 (52.5)	13 (21.7)				
Instability	MSS	12 (30.0)	19 (31.7)	0.8	1.583	0.769 -3.262	0.2
	MSIL	26 (65.0)	35 (58.3)	0.2	1.346	0.810 - 2.236	0.3
	MSIH	2 (5.0)	6 (10.0)	0.1	3	0.606 - 14.864	0.2

Studied microsatellites are represented as Frequency and percent; the data were analyzed by X^2^ test. OR; Odd Ratio, C.I; Confidence Interval, p value calculated depend on logistic regression analysis. * P value <0.05 is significant, ** P value <0.01 is highly significant.

**Table 6:** Studied Microsatellites Regarding the Stage of Disease with the Risk Assessment in Plasma

		Fibrosis	Cirrhosis	P. Value	OR	95% C.I	P. Value
		N=40 (%)	N=60 (%)				
BAT-25	Stable	23 (57.5)	54 (90.0)	0.001**	0.4	0.27 - 0.61	0.001**
	Instable	17 (42.5)	6 (10.0)				
BAT-26	Stable	22 (55.0)	48 (80.0)	0.01*	0.52	0.33 - 0.82	0.01*
	Instable	18 (45.0)	12 (20.0)				
NR-21	Stable	21 (52.5)	48 (80.0)	0.001**	0.5	0.32 - 0.78	0.001**
	Instable	19 (47.5)	12 (20.0)				
NR-22	Stable	24 (60.0)	48 (80.0)	0.03*	0.58	0.37 - 0.92	0.03*
	Instable	16 (40.0)	12 (20.0)				
NR-24	Stable	27 (67.5)	42 (70.0)	0.5	0.93	0.56 - 1.55	0.8
	Instable	13 (32.5)	18 (30.0)				
D-8S1771	Stable	23 (57.5)	54 (90.0)	0.001**	0.4	0.27 - 0.61	0.001**
	Instable	17 (42.5)	6 (10.0)				
D-8S277	Stable	26 (65.0)	40 (66.7)	0.5	0.96	0.58 - 1.58	0.9
	Instable	14 (35.0)	20 (33.3)				
D-8S298	Stable	24 (60.0)	51 (85.0)	0.001**	0.5	0.32 - 0.78	0.001**
	Instable	16 (40.0)	9 (15.0)				
D-2S123	Stable	23 (57.5)	36 (60.0)	0.5	0.94	0.58 - 1.53	0.8
	Instable	17 (42.5)	24 (40.0)				
D-17S250	Stable	26 (65.0)	40 (66.7)	0.5	0.96	0.58 - 1.58	0.9
	Instable	14 (35.0)	20 (33.3)				
Instability	MSS	4 (10.0)	12 (20.0)	0.05*	3	0.968 - 9.302	0.05*
	MSIL	13 (32.5)	31 (51.7)	0.01*	2.4	1.248 - 4.557	0.01*
	MSIH	23 (57.5)	17 (28.3)	0.2	0.74	0.395 - 1.383	0.3

Studied microsatellites are represented as Frequency and percent; the data were analyzed by X^2^ test. OR; Odd Ratio, C.I; Confidence Interval, p value calculated depend on logistic regression analysis. * P value <0.05 is significant, ** P value <0.01 is highly significant.

**Table 7:** The Associations between the Microsatellite Instability Level with the Studied Parameters

		In Tissues			In plasma		
		MSI-L	MSI-H	P. Value	MSI-L	MSI-H	P. Value
		N=61	N=8		N=44	N=40	
Age		49.8±10.4	50.9±3.4	0.6	48.2±9.1	51.2±9.5	0.1
Sex	Female	10 (16.4)	3 (37.5)	0.2	9 (20.5)	9 (22.5)	0.7
	Male	51 (83.6)	5 (62.5)		35 (79.5)	31 (77.5)	
ALT		77.0±36.7	77.3±18.3	0.9	60.5±15.1	91.3±38.2	0.001**
AST		84.1±43.1	79.6±9.7	0.5	70.0±0.01	70.0±0.01	0.9
Alb		1.8±1.1	0.8±0.2	0.001**	2.0±0.9	1.3±0.9	0.01*
Bilirubin		2.7±1.2	2.6±0.4	0.7	2.7±1.0	2.5±1.2	0.4
Alpha feto-protein		290.0 (25.0- 1,075.0)	1300.0 (290.0- 2575.0)	0.01*	80.0 (42.5- 297.5)	950.0 (50.0- 2300.0)	0.001**
S-creatinine		1.1±0.4	1.3±0.3	0.06	1.1±0.4	1.3±0.3	0.06
No. of masses		1.7±1.0	2.9±0.8	0.01*	1.1±0.3	2.4±1.0	0.001**
Tumor size		3x1.5 (1.5x1.5 – 2.5x2.5)	2x1.5 (1x1.5 – 11x10)	0.04*	3.0X1 (1X1.2- 2.5X1.5)	5.9X1 (4.5X1- 7X9)	0.001*
Tumor Grade	I	32 (52.5)	0 (0.0)	0.001**	34 (77.3)	12 (30.0)	0.001**
	II	14 (23.0)	3 (37.5)	0.04*	10 (22.7)	8 (20.0)	0.5
	III	15 (24.6)	5 (62.5)	0.01*	0 (0.0)	20 (50.0)	0.001**
Pattern	Acinar	24 (39.3)	0 (0.0)	0.001**	24 (54.5)	10 (25.0)	0.001**
	Solid	23 (37.7)	5 (62.5)	0.02*	17 (38.6)	16 (40.0)	0.8
	Acinar/ Solid	14 (23.0)	3 (37.5)	0.04*	3 (6.8)	14 (35.0)	0.001**
Steatosis		0.03 (0.02- 0.1)	0.02 (0.02- 0.5)	0.7	0.03 (0.02- 0.06)	0.03 (0.02- 0.1)	0.6
HAI	A1	10 (16.4)	3 (37.5)	0.01*	8 (18.2)	6 (15.0)	0.4
	A2	37 (60.7)	5 (62.5)	0.7	36 (81.8)	20 (50.0)	0.001**
	A3	14 (23.0)	0 (0.0)	0.01*	0 (0.0)	14 (35.0)	0.001**
Hepatomegaly	Positive	37 (60.7)	5 (62.5)	0.6	3 (6.8)	37 (92.5)	0.001**
Ascites	Positive	39 (63.9)	5 (62.5)	0.6	24 (54.5)	36 (90.0)	0.001**
Splenomegaly	Positive	37 (60.7)	8 (100.0)	0.03*	20 (45.5)	34 (85.0)	0.001**
Edema Lower Limbs	Positive	40 (65.6)	8 (100.0)	0.04*	12 (27.3)	40 (100.0)	0.001**

Age, ALT, AST, Alb, Bilirubin, S-creatinine, and No. of masses are represented as Mean and SD; the data were analyzed by t test, But Alpha feto-protein, Tumor size, and Steatosis are represented as Median and Interquartile Range (IR) (25% -75); the data were analyzed by Mann-Whitney U test., While Sex, Tumor Grade, Pattern, HAI, Hepatomegaly, Ascites, Splenomegaly, and Edema Lower Limbs are represented as Frequency and percent; the data were analyzed by X^2^ test. * P value <0.05 is significant, ** P value <0.01 is highly significant.

## Discussion

Hepatocellular carcinoma is the third deadliest and fifth most common cancer worldwide (Yang et al., 2010). It is considered one of the most violent diseases in the world (Chang-Hao et al., 2015) and is a major health problem in Egypt representing 13% of all cancers in Egypt (Zeeneldin et al., 2015). In Egypt, according to the study carried out by the National Population-Based Cancer Registry Program, in 2014, liver cancer was ranked first, among cancers in Egyptian males (33) and females (13.5) (Ibrahim et al., 2014). HCC is associated with a background of chronic and persistent infection of hepatitis B virus (HBV) or hepatitis C virus (HCV) (Caccamo et al., 2014). Data collected from the patients’ medical files showed that they were HCV genotype 4, that was detected by RT- PCR, at our laboratory, at TBRI, according to the method documented by (Shemis et al., 2012).

Biomarkers are beneficial in the detection of cancer at an early stage which is important since early detection has a direct effect on clinical outcomes and prognosis. Serum alpha-fetoprotein (AFP) is a useful tumor marker for the detection and monitoring of HCC, but unfortunately the false negative rate with AFP level alone may be as high as 40% for patients with early stage HCC (Nobuhiro et al., 2015 ; Samah et al., 2017b), therefore, it is important to identify other molecular markers for the diagnosis of the disease.

Microsatellite instability have been studied in many cancers such as colorectal cancer, melanoma, endometrial carcinoma, breast cancer, bladder cancer and hepatocellular carcinoma. In the present work we studied 5 monomorphic repeats (BAT25, BAT26, NR21, NR22, NR24) and 5 highly polymorphic repeats (D-8S298, D-8S1771, D-8S277, D-2S123 and D-17S250). Among the 100 HCC tumors analyzed, 8% considered as displaying a typical MSI-H phenotype as defined by instability in at least 3 of the 10 repeats analyzed, 61% tumors displayed MSI-L and 31% displayed MSS. While in serum the instability was, 40% for MSI-H, 44% for MSI-L and 16% for MSS.

Microsatellite instability testing is most commonly performed via PCR analysis of tumor tissue and adjacent nontumor specimens. In our study, the use of corresponding blood samples from all patients for MSI analysis is unique and the results are considered a good addition in this field.

Our results for tissues showed the instability according to the source of viremia, Interestingly, a percentage of 36.4 was observed representing MSI-H in cases of HBV infection (P. value <0.001), while 37.2% was MSS in cases of HCV infection (P. value <0.001). These results are in agreement with the findings of Salvucci et al., 1996 who concluded a close association between genomic instability and cirrhosis linked to hepatitis B viral infection (P<0.01). Our results for plasma samples regarding the source of viremia indicate that, 4 MS were observed to have a clear instability in cases of HBV infection than HCV which were, BAT-26, D-8S277, D-2S123 and D-17S250.

In the present study, 5 of the 10 studied repeats had instability in cirrhotic livers more than fibrotic, this result is similar to Claire et al., 2017 who reported that the levels of MSI tended to be higher in patients with cirrhosis and also in accordance with the results of Kondo et al., 2000 who found that MSI in cirrhotic liver was almost three times higher than in liver with chronic hepatitis. In contrast, many other studies from Italy, (Ronchalli et al., 2000) Taiwan (Sheu et al., 1999) Japan (Saeki et al., 2000) South Africa (Martins et al., 1999) and the USA (Wang et al., 2001) found little evidence of MSI in HCC of patients with chronic liver disease.

Our results regarding the stage of the disease of plasma samples proved that 6 MS were observed to have a clear instability in cases of fibrosis grade than cirrhosis which were, BAT-25, BAT-26, NR-21, NR-22, D-8S1771 and D-8S298, these results prove the importance of using these loci for verification of liver fibrosis.

Claire et al study population consisted of 122 patients with histologically-proven HCC, he carried out using 5 quasi-monomorphic mononucleotide repeats markers co-amplified in a single pentaplex PCR reaction , and 13 highly polymorphic dinucleotide repeat markers, Abnormal profiles consisting in the insertion or deletion of few nucleotides have been observed in 8 tumors (1 at NR21, 1 at BAT26, and 6 at BAT25) this is on contrary to our results which reported that no instability was found at BAT25. The authors have also screened for instability at 13 dinucleotide repeated loci, The majority of samples (90/122) displayed no detectable instability at any of the 13 dinucleotide repeat loci analyzed. At maximum, 3 abnormal profiles could be detected in 3 tumors , which is not enough to classify these tumors as MSI-H (Claire et al., 2017). 

One hundred and sixty-four patients with HCC affecting non-cirrhotic livers were operated and only 37 patients were selected for low alcohol consumption and absence of HBV or HCV infection. MSI was studied in 37 hepatocellular carcinomas by use of the panel of five microsatellite markers described by the consensus conference workshop in 1998 and five others that have already been used to detect MSI in HCC and other tumor types. The results showed Six of the 37 (16) tumors were MSI-H, 10 (27) were MSI-L and 21 (57) were MSS (Chiappini et al., 2004). These results did not match to ours which stated that the percentage of instability displaying MSI-L is 61% while that of MSS is 31%.

Thirty seven HCC patients who underwent liver resection were included in a study for Tongi et al., 2009, normal and tumor DNA was extracted, MSI analysis was performed using a panel of monomorphic microsatellites markers: BAT-25, BAT-26, NR21, NR24 and NR27 (Loukola et al., 2001; Umar et al., 2004). No differences between microsatellite lengths as assessed in PCR products were found for any of the five microsatellite markers in any patient. These findings provided no evidence for MSI.

For tissue samples, the present study showed a significant association between the MSI-H phenotype instability and high level of AFP (P. <0.01), higher number of tumor masses (P. <0.01), larger tumor size (P. =0.04), advanced tumor grade II (P. =0.04) and grade III (P. <0.01), solid pattern (P. =0.02) and acinar/solid pattern (P. =0.04), HAI A1 (P. <0.01), Splenomegaly (P. =0.03), and in patients with lower limb edema (P. =0.04).

For plasma samples, the results showed a significant association with high level of ALT (P. value <0.001), low albumin (P. value =0.01), high level of AFP (P. value <0.01), higher number of masses (P. value <0.001), larger tumor size (P. value <0.001), advanced tumor grade III (P. value <0.001), Acinar/Solid pattern (P. value <0.001), HAI A3 (P. value <0.001), Hepatomegaly (P. value <0.001), Ascites (P. value <0.001), Splenomegaly (P. value <0.001), and in patients with lower limb edema (P. value <0.001).

In a conclusion, our findings could point to the achievement that HCC patients could be diagnosed by MSI analysis using blood sample as non-invasive way by PCR, this method may give more rapid and accurate results and this conclusion highlights the importance of using MSI as routine assessment in clinical fields. 

## Author Contribution Statement

None.
